# Similar Levels of Efficacy of Two Different Maintenance Doses of Adalimumab on Clinical Severity and Quality of Life of Patients with Hidradenitis Suppurativa

**DOI:** 10.3390/jcm11144037

**Published:** 2022-07-12

**Authors:** Luca Fania, Giulia Giovanardi, Tonia Samela, Dante Caposiena, Andrea Chiricozzi, Flaminia Antonelli, Pierluigi Saraceni, Fulvia Elia, Simone Garcovich, Davide Ciccone, Maria Vittoria Cannizzaro, Emanuele Miraglia, Chiara Iacovino, Sandra Giustini, Nevena Skroza, Alessandra Mambrin, Concetta Potenza, Luca Bianchi, Ketty Peris, Damiano Abeni

**Affiliations:** 1 Clinical Epidemiology Unit and Dermatology Department, Istituto Dermopatico dell’Immacolata, IDI-IRCCS, 00167 Rome, Italy; l.fania@idi.it (L.F.); giovanardigiulia1@gmail.com (G.G.); t.samela@idi.it (T.S.); d.ciccone@idi.it (D.C.); d.abeni@idi.it (D.A.); 2 Department of Dermatology, Università Cattolica del Sacro Cuore, Fondazione Policlinico Universitario A Gemelli IRCCS, 00168 Rome, Italy; andrea.chiricozzi@unicatt.it (A.C.); flaminiantonelli@gmail.com (F.A.); ketty.peris@unicatt.it (K.P.); 3 Department of Dermatology, University of Rome “Tor Vergata”, 00133 Rome, Italy; dcaposienacaro@gmail.com (D.C.); mariavittoria.cannizzaro@gmail.com (M.V.C.); luca.bianchi@uniroma2.it (L.B.); 4 Istituto Dermatologico San Gallicano, IRCCS, 00144 Rome, Italy; pierluigi.saraceni@ifo.gov.it (P.S.); fulviaelia@gmail.com (F.E.); 5 Dermatology Clinic Umberto I, Sapienza University of Rome, 00185 Rome, Italy; emanuele.miraglia@hotmail.it (E.M.); chiara.iacovino86@gmail.com (C.I.); sandra.giustini@uniroma1.it (S.G.); 6 Dermatology Unit, Department of Medical and Surgical Sciences and Biotechnologies, Daniele Innocenzi, Sapienza University of Rome—Polo Pontino, 00185 Rome, Italy; nevena.skroza@uniroma1.it (N.S.); mambrinalessandra@gmail.com (A.M.); concetta.potenza@uniroma1.it (C.P.)

**Keywords:** hidradenitis suppurativa, acne inversa, verneuil disease, adalimumab, patient-reported outcome measures, quality of life, efficacy, pain

## Abstract

Adalimumab is the only biologic agent approved for the treatment of moderate-to-severe hidradenitis suppurativa (HS) patients (i.e., with Hurley II or III), which is recommended in two different maintenance doses (i.e., 40 mg weekly or 80 mg every two weeks). We conducted a prospective multicentric study to measure outcomes related to the severity of disease and quality of life (QoL) of patients affected by moderate-to-severe HS, treated with adalimumab at a maintenance dosing of 40 mg or 80 mg. Assessments were performed at baseline (T0) and after 32 weeks of treatment (T32). We enrolled 85 moderate-to-severe HS Italian patients, 43 men (50.6%) and 42 women, aged between 16 and 62 years (median 31 years, interquartile range 24.4–43.8). Statistically significant improvements were observed for clinical status (with a mean reduction of 7.1 points for the International Hidradenitis Suppurativa Severity Score System (IHS4)), pain levels (3.1 mean decrease in VAS), and QoL (3.4 mean improvement in DLQI score). Patients with no comorbidities, and those with higher levels of perceived pain showed significantly greater improvement in QoL than their counterpart from T0 to T32. As for the proportion of patients who at follow-up reached the minimal clinical important difference (MCID) in QoL, significantly higher proportions of success were observed for age (patients in the 29–39 category), pain (patients with higher reported pain), and Hurley stage III. While both treatment regimen groups (i.e., 40 vs. 80 mg) improved significantly, no statistical differences were observed when comparing the two treatment dosages.

## 1. Introduction

Hidradenitis Suppurativa (HS) is a chronic, recurrent, and debilitating skin condition of the hair follicle [1,2] characterized by painful and suppurating lesions mainly localized in the apocrine gland-bearing areas. The worldwide prevalence of HS has been estimated to be from <0.05% to >4% [3], although in Europe and United States, the overall prevalence is around 1% [4,5,6]. Regarding the incidence, it has been estimated around 11.4 cases per 100000 person-years in the United States, with a much higher risk for women than men (16.1 vs. 6.8) [7]. HS is typically a multifactorial disease, although currently, it is considered an auto-inflammatory disease [8,9]. Several cytokines such as TNF- α, IL-1, IL-17 and IL-23 are involved in HS pathogenesis, and their blockade could be a rational therapeutic approach. Currently, TNF inhibition with adalimumab is the only biologic agent approved by the FDA and EMA for the treatment of moderate-to-severe HS patients [10]. As described by the International HS guidelines [1], US Food and Drug Administration (FDA) [11], and the European Medicines Agency (EMA) [12], adalimumab is recommended at the initial dose of 160 mg, followed by 80 mg at week 2 and 40 mg weekly from week 4 and thereafter, or, alternatively, 80 mg every two weeks. It has been reported that adalimumab dose intensification with 80 mg/week favored an enhanced level of effectiveness considering the IHS4 score, pain index, HS-physician global assessment, pain, and Cardiff dermatology life quality index for most HS patients [13,14]. Furthermore, HS is associated with high expression levels of interleukin (IL)-17A in the bloodstream [15,16] and in the skin-sites affected by lesions [17].

The aim of this study was to compare the clinical severity, quality of life (QoL), and the perceived pain due to HS, at baseline and after 32 weeks, in Italian patients affected by moderate-to-severe HS (Hurley II and III) and treated with two different adalimumab regimens (i.e., maintenance therapy with 40 mg weekly or 80 mg each two weeks).

## 2. Materials and Methods

### 2.1. Study Design

This is a prospective multicentric study, in which we measured specific outcome values related to the severity of disease and QoL of patients affected by HS treated as maintenance therapy with adalimumab in two different dosages, i.e., 40 mg weekly or 80 mg each two weeks. Scores were registered at baseline (T0) and at follow-up, after 32 weeks of treatment (T32). The study was approved by the Institutional Ethical Committee of IDI-IRCCS, Rome, with the number 607/1, approved on 17 September 2019.

### 2.2. HS Patients and Measures

Consecutive patients with a diagnosis of HS were recruited from October 2019 to October 2020, from five different centers (IDI-IRCCS, University of Cattolica Sacro Cuore—Fondazione Policlinico Universitario A. Gemelli, University of Rome ‘Tor Vergata’, San Gallicano Dermatological Institute IRCCS, Sapienza University of Rome, and Sapienza University of Rome—Polo Pontino). The inclusion criteria were: (1) age 16 years or more; (2) female or male sex; (3) patients under treatment with adalimumab (40 mg or 80 mg dosage) and treated with topical therapy; (4) diagnosis of moderate-to-severe HS (Hurley II and III); (5) written informed consent, signed by the patient.

The exclusion criteria were: (1) presence of nodular acne associated with macro-comedones, single pilonidal cysts, and recurrent necrotic folliculitis of the scalp in absence of other criteria to fulfil the diagnosis of HS [18]; (2); HS in treatment with other drugs different from adalimumab.

At baseline, each patient filled out a standardized sociodemographic data collection form concerning: age; sex; weight and height, BMI; age at HS onset and age at diagnosis; comorbidity (i.e., a disease or medical condition that is simultaneously present with HS in the patient); and smoking habit (number of cigarettes per day). Complete socio-demographic information is included in Table 1. Then, the patient was asked to complete the Dermatology Life Quality Index (DLQI) and the Visual Analogue Scale (VAS) regarding the severity of pain due to HS. During the visit, the dermatologist collected information regarding the Hurley stage, number of body lesions (nodules, abscesses, fistulas, and sinus tracts), the International Hidradenitis Suppurativa Severity Score (IHS4), and the Physician Global Assessment (PGA) in order to assess the clinical severity of HS, and the assigned dosage of adalimumab. At the 32-week follow-up (T32), patients completed a new questionnaire containing DLQI and VAS, and the dermatologist computed the Hidradenitis Suppurativa Clinical Response (HiSCR), in addition to re-scoring the IHS4 and the PGA.

### 2.3. Physician-Assessed Symptoms’ Severity

The physician assessment was conducted through the IHS4 (i.e., score of 3 or less indicates mild HS, a score of 4–10 moderate HS, and a score of 11 or more severe HS [19]); the Hurley classification (i.e., Hurley stage I; Hurley stage II or Hurley III [20]); the Physician Global Assessment (PGA) (i.e., a 5-point scoring system used to assess disease severity [21]); the HiSCR (defined as a ≥50% reduction in inflammatory lesion count, and no increase in abscesses or draining fistulas when compared with baseline); and the minimal clinically important difference (MCID) (derived from the DLQI and measure the responsiveness to change in inflammatory skin diseases [22].

### 2.4. Patient Reported Qol Outcome Measures 

In order to assess the patient’s QoL, the following tools were used: VAS (used to achieve a statistically measurable and reproducible classification of symptom severity and disease control [23]); the DLQI (a useful tool to assess the symptoms, feelings, daily activities, leisure, work, and school, personal relationships, and treatment of patients; the higher the score, the greater the impairment of QoL [24]).

### 2.5. Statistical Analysis 

All analyses were performed using SPSS 25.0. The convergence and dispersion trends for quantitative variables were expressed as mean ± standard deviation, and qualitative variables were expressed as frequencies and percentages. Differences among DLQI scores were obtained through the comparison between baseline and T32, and results have been identified with ΔDLQI and used as the first QoL outcome measure, as well as the percentage of patients who reached the MCID (%MCID). The percentage of HS patients who achieved the HiSCR at week 32 has been reported in Table 1. All socio-demographic and clinical variables were recorded in categories, and for each level of the variables of interest we computed the ΔDLQI and %MCID. The normality of the frequency distributions of the main variables of interest was tested using the Kolmogorov–Smirnow test. As none of the deltas of interest (i.e., for IHS4, DLQI, VAS, and PGA) between T0 and T32 were normally distributed, we adopted nonparametric tests. Therefore, for independent samples (Table 1) the Mann–Whitney U-test was used for variables with two levels, while the Kruskal–Wallis test for independent variables was used for three or more levels. For two matched samples (Table 2), we used the Wilcoxon signed-rank test. For the categorical variables (i.e., % reaching MCID or HiSCR) the chi-square test was used. 

Finally, to assess the possible independent association of the main variables of interest with the %MCID, while simultaneously controlling for the main potential confounding variables, we performed a logistic regression analysis. In addition to the main exposure of interest (i.e., dose regimen of adalimumab), we included in the model sex and gender by default; furthermore, the variables that in univariate analysis had *p* < 0.150 (model described in Table 3).

## 3. Results

### 3.1. Patient Population

There were 107 patients enrolled at baseline, and 22 (20.6%) did not reach week 32. At baseline, there were no differences between the patients who completed the follow-up and those who did not. The IHS4 was 19.77 in the “lost to follow-up” group and 22.16 in the “compliant” group (*p* = 0.488); the PGA was 4.09 in both groups (*p* = 0.988); the DLQI scores were 15.27 and 15.14 in the two groups, respectively (*p* = 0.940); and the VAS scores were 5.45 in the non-compliant group and 6.22 in the compliant group (*p* = 0.185). The study population therefore included 85 patients with HS. Among them, 43 were men (50.6%) and 42 women, aged between 16 and 62 years (mean = 33.8; SD ± 11.9). The sociodemographic features of the sample, QoL and clinical differences at T32 are reported in Table 1. 

Most of the patients (50/85, 58.8%) had a BMI > 25, and 61/85 (71.8%) were smokers or ex-smokers. Sixteen of 85 (18.8%) patients presented n IHS4 between 5 and 10 (moderate HS) while 69 had a IHS4 > 11 (severe HS). Sixty-two patients (72.9%) were treated with adalimumab 40 mg while 23 (27.1%) with adalimumab 80 mg. 

### 3.2. Treatment-Related Outcome Variables 

In the main variables of interest, when looking at the mean differences of DLQI scores between T0 and T32 (Table 1), we observed significantly higher levels of improvement in older patients (*p* = 0.021) and in those with higher pain VAS scores at baseline (*p* = 0.016,). Patients with no comorbidities (vs. patients who had at least one) also had a higher level of improvement, but it did not reach statistical significance (*p* = 0.063).

As for the proportion of patients who at follow-up reached the MCID in QoL, higher proportions of success were observed for age (patients in the 29–39 category), pain (patients with higher reported pain), and Hurley stage III. A difference was also observed for BMI, with a greater proportion of MCID achievement for normal weight patients, though it did not reach statistical significance. However, no significant differences in HiSCR were observed in any of the variables of interest.

Table 2 shows the baseline–T32 mean differences in outcome of the IHS4, DLQI, VAS, and PGA. All these outcomes of interest showed a highly statistically significant improvement from baseline to T32. When stratifying by treatment dosage, no statistical differences were observed, although in the clinical measures computed by the dermatologists the mean improvement in IHS4 was 7.8 for the 40 mg and 5.1 for the 80 mg.

### 3.3. Predictive Factors for Treatment Response

Table 3 shows the results of the logistic regression analysis. The odds of reaching the MCID were significantly higher for patients in the 29–39-year-old group (adjusted Odds Ratio (adjOR) 6.1 vs. the 16–28-year-olds), for those with a BMI within the norm (adjOR 4.3 vs. overweight and obese patients), and those with more severe pain (adjOR 3.5 for patients with VAS values ≥7 vs. < 7). In addition, males had an adjOR of 2 compared to females to attain the MCID, though this difference did not reach statistical significance. 

## 4. Discussion

HS is a debilitating disease characterized by significant chronic inflammation and compromised QoL [25]. So far, no studies have analyzed the efficacy and QoL indexes in patients with moderate-to-severe HS treated with adalimumab 40 mg weekly or 80 mg every two weeks, although some encouraging results regarding long-term use of adalimumab were reported by Hafner et al. (2021). These authors, in fact, observed a significant improvement from baseline to week 52 of treatment with adalimumab in DLQI and other QoL measures [26,27].

In our sample, regarding the differences of DLQI scores considering T0 and T32, a higher level of improvement was reported in patients with no comorbidities and in those with higher pain score. This could be because patients with comorbidities could present other diseases that did not respond to anti-TNF therapy. Otherwise, patients with a higher pain score could improve with biologic therapy due to the immunomodulation of the immune system and to the anti-inflammatory effects of this therapy that, consecutively, lead to an improvement of DLQI. Furthermore, one center showed a significantly higher QoL improvement, probably due to selection of patients with higher severity and pain.

Regarding patients who, at follow-up, reached the MCID in QoL, a significant higher proportion of success was reported for age in the “29–39 group”, patients with higher reported pain, and those affected by Hurley stage III. Our data diverge from a recent multicenter cohort study that analyzed the clinical and QoL parameters predicting response in 389 patients with HS treated with adalimumab 40 mg weekly as maintenance. In this study, none of the parameters of sex, BMI, age at onset, age at diagnosis, age at baseline, HS phenotypes, or smoking correlated with response to adalimumab; otherwise, the therapeutic delay was identified as a significant risk factor for nonresponse to adalimumab. Furthermore, in our study, a large difference was also observed for BMI, with a greater proportion of response for normal weight patients, even if it did not reach statistical significance. Moreover, no significant statistical differences in HiSCR were reported in the considered variables of interest. This could depend on the sample size and, therefore, did not reveal a significant difference in the efficacy of the treatment with the two different dosages assessed with the HiSCR outcome.

Considering IHS4, DLQI, VAS, and PGA, all these outcomes presented a statistically significant improvement from baseline to T32 in the two main group of our study (i.e., 40 vs. 80 mg); however, no statistical differences were reported subsequently to the stratification by treatment dosage. Specifically, there was a mild higher improvement in IHS4 for the group treated with 40 mg compared to 80 mg. 

Regarding the logistic regression analysis, the odds of reaching the MCID were significantly higher for patients in the 29–39-year-old group, for those with normal BMI, and those with more severe pain. Furthermore, males had a higher odds of reaching MCID compared to females, but this difference did not reach statistical significance.

Depression is a well-established and common comorbidity in patients with HS and is generally associated with a worse clinical outcome [28,29]. The existing literature indicates that acute and chronic pain, mood and anxiety disorders, and disability all contribute to poor QoL in individuals with HS [30,31]. The unforeseeable effect of painful, malodorous abscesses seems to led to feelings of helplessness and hopelessness in individuals with HS, who avoid social interactions to cope with the disease and reduce the embarrassment; this chain of events, in turn, may contribute to depressive symptoms [32]. Even chronic pain due to HS lesions may contribute to the onset or may aggravate existing signs of depressive symptoms [33]. Moreover, an HS registry-based study with a larger sample size (*n* = 3207) found that an anxiety disorder was diagnosed in 3.9% of those with HS, compared to 2.4% of those without HS [33]. In addition, a systematic review and meta-analysis of 10 studies comprising 40,307 participants with HS found a prevalence of anxiety of 4.9% [29]. Not specifically considering anxiety and depression, which also contribute to the worsening, even when no psychiatric conditions are officially diagnosed, the QoL in patients with HS is severely impaired. Sampogna et al. (2020) noticed that this impairment may exceed that of other burdensome skin diseases, such as psoriasis, neurofibromatosis, chronic urticaria, and atopic dermatitis [34]. Even if the disease was clinically ‘mild/moderate’ according to the IHS4 severity scale, QoL mental dimensions scores in HS patients were very similar to those of patients with diagnosed psychiatric conditions [34].

## 5. Conclusions

The main finding of our multicenter prospective study, conducted within a cohort of 85 patients with moderate-to-severe HS treated with adalimumab 40 mg and 80 mg as maintenance, is that both regimens showed comparable levels of clinical efficacy and QoL enhancement, with a significant improvement of the HS clinical manifestations, but no statistical differences were observed when comparing the two treatment dosages. Therefore, the choice between the two treatment regimens should take into particular account patient preferences (such as a reduced number of injections), as well as the doctor’s perception of the patient’s ability to adhere to treatment prescriptions.

## Figures and Tables

**Table 1:** Sociodemographic and clinical features of the sample at baseline. Quality of life and clinical differences at T32 for the baseline variables of interest.

VAR	LEV	%		Δ DLQI			MCID		HiSCR	
		*N* *	**%**	**M**	**SD**	***p***	**%**	***p***	**%**	***p***
Overall	/	85	/	3.4	4.4	/	41.2	/	24.7	
sex	M	43	50.6	3.4	4.3		46.5		37.2	
	F	42	49.4	3.3	4.5	0.915	35.7	0.313	11.9	0.007
Age	16–28	34	40.0	2.1	3.9		23.5		32.4	
	29–39	22	25.9	4.3	4.1		59.1		22.7	
	≥40	28	34.1	4.4	5.0	0.021	50.0	0.017	17.9	0.406
Age of onset	11–16	38	44.7	3.2	4.9		39.5		21.1	
	17–28	38	44.7	3.7	3.9		44.7		31.6	
	≥29	8	10.6	3.0	4.6	0.613	37.5	0.896	12.5	0.395
BMI	<25	35	41.2	4.2	4.5		51.4		28.6	
	˃25	50	58.8	2.8	4.3	0.105	34.0	0.108	22.0	0.489
Comorbidities	yes	49	57.6	2.5	3.9		36.7		27.8	
	no	36	42.4	4.6	4.8	0.063	47.2	0.332	22.4	0.574
Smoker	Never	24	28.2	2.8	4.5		29.2		25.0	
	ex	61	71.8	3.6	4.4	0.314	45.9	0.158	24.6	0.969
IHS4	MOD (5–10)	16	18.8	3.0	4.7		43.8		31.3	
	SEV (˃11)	69	81.2	3.5	4.4	0.905	40.6	0.816	23.2	0.501
VAS	0–6	40	47.1	2.0	3.4		30.0		30.0	
	≥7	45	52.9	4.6	4.8	0.016	51.1	0.048	20.0	0.286
Hurley	2	45	52.9	2.8	4.1		31.1		24.4	
	3	40	47.1	4.1	4.7	0.193	52.5	0.046	25.0	0.953
ADA Dose	40	62	72.9	3.4	4.5		38.7		25.8	
	80	23	27.1	3.2	4.3	0.988	47.8	0.448	21.7	0.699
Diagnostic delay (years)	0–2	27	32.1	3.2	4.3		37.0		22.2	
	3–7	29	34.5	3.5	4.0		48.3		31.0	
	≥8	28	33.4	3.6	5.0	0.821	39.3	0.662	21.4	0.649
Center	1	10	11.8	6.8	6.8		60.0		10.0	
	2	5	5.9	8.4	2.7		100.0		80.0	
	3	19	22.3	0.5	2.2		5.3		0.0	
	4	46	54.1	2.8	3.5		39.1		32.6	
	5	5	5.9	7.8	2.3	<0.001	100.0	<0.001	20.0	0.002

* Totals may vary because of missing data. For the comparison of the continuous variable (i.e., ∆ DLQI) the Mann–Whitney U-test was used for variables with two levels, while the Kruskal–Wallis test for independent variables was used for three or more levels. For the categorical variables (i.e., % reaching MCID or HiSCR) the chi-square test was used.

**Table 2:** Mean improvement from baseline to follow-up (T0-T32) for clinical and patient-reported outcome measures.

				CI 95%				
Outcome Variable	M	SD	SE	Lower	Upper	t	df	*p*
IHS4 (T0/T32)	7.11	9.76	1.06	5.00	9.21	6.71	84	<0.001
PGA (T0/T32)	0.75	0.86	0.03	0.57	0.94	8.09	84	<0.001
DLQI (T0/T32)	3.38	4.40	0.48	4.33	2.43	−7.07	84	<0.001
VAS (T0/T32)	3.13	2.36	0.26	3.64	2.62	−12.23	84	<0.001

M = mean; SD = standard deviation; SE = standard error; CI 95% = 95% confidence interval; df = degrees of freedom. *p*-values are from the Wilcoxon signed-rank test for two matched samples.

**Table 3:** Multiple logistic regression models: relative odds of reaching the MCID at T32.

Predictor Variable		adjOR	95% CI	*p*
Sex	Female	1		
	Male	2.0	0.7–5.7	0.202
Age	16–28	1		
	29–39	6.1	1.6–22.9	0.007
	≥40	2.3	0.7–8.1	0.194
BMI	˃25	1		
	≤25	4.3	1.4–13.4	0.013
HURLEY stage	2	1		
	3	2.1	0.7–5.9	0.182
VAS	<7	1		
	˃7	3.5	1.1–11.1	0.031
ADA dose	40 mg	1		
	80 mg	1.4	0.5–4.3	0.566

## Data Availability

The data presented in this study are available on reasonable request from the corresponding author.
